# Attitudes of healthy volunteers to genetic testing in phase 1 clinical trials

**DOI:** 10.12688/f1000research.26828.1

**Published:** 2021-03-30

**Authors:** Sebastian Levesque, Thomas M. Polasek, Eric Haan, Sepehr Shakib

**Affiliations:** 1 Department of Clinical Pharmacology, Royal Adelaide Hospital, Adelaide, South Australia, 5000, Australia; 2 Discipline of Pharmacology, The University of Adelaide, Adelaide, South Australia, 5000, Australia; 3 Centre for Medicines Use and Safety, Monash University, Melbourne, Victoria, 3052, Australia; 4 Certara, Princeton, New Jersey, 08540, USA; 5 Adult Genetics Unit, Royal Adelaide Hospital, Adelaide, South Australia, 5000, Australia; 6 Faculty of Health and Medical Science, The University of Adelaide, Adelaide, South Australia, 5000, Australia

**Keywords:** phase 1, genetics, healthy volunteers, informed consent, pharmacogenetics

## Abstract

**Background:** Genetic testing in clinical trials introduces several ethical and logistical issues to discuss with potential participants when taking informed consent. The aim of this study was to explore the attitudes of healthy volunteers in phase 1 studies to the topics of genetic security, genetic privacy and incidental genetic findings.

**Methods:** Healthy volunteers presenting for screening appointments at a phase 1 clinical trial unit (CMAX Clinical Research, Adelaide, Australia) took an anonymous paper survey about genetic testing.

**Results:** There were 275 respondents to the survey. The mean age was 27 years (range 18-73); 54% were male and 53% were of North/Western European ethnicity. Just over half the healthy volunteers thought genetic security (56%) and genetic privacy (57%) were “important” or “very important”. However, the security of their genetic information was ranked less important than other personal information, including mobile phone number, internet browser search history and email address. Two-thirds of respondents would trade genetic privacy for re-identifiability if information relevant to their health were discovered by genetic testing. Healthy volunteers favoured the return of incidental genetic findings (90% indicated this was “important” or “very important”). A level of risk (10 to 90%) for developing a serious medical condition that would “trigger” the return of incidental genetic findings to participants was not identified.

**Conclusions:** Healthy volunteers screening for phase 1 clinical trials have mixed views about the importance of genetic security and genetic privacy, but they strongly favour the return of incidental genetic findings that could affect their health. These issues should be discussed with potential participants during informed consent for phase 1 clinical trials with genetic testing.

## Introduction

Genetic testing during clinical drug trials can determine whether genetics influences how patients respond to drug therapy. ^1, 2^ Increasingly, genetic information is collected from healthy volunteers during phase 1 clinical studies to identify factors that inform later stages of drug development, including dosing strategies, patient selection, and the potential role of biomarkers to monitor drug responses. ^1^

Genetic testing in clinical trials introduces several ethical and logistical issues for participation, including concerns about genetic security and genetic privacy and how investigators should proceed with incidental genetic findings. Genetic security is the secure storage of genetic data and material. Clinical trial facilities and pharmaceutical companies must store these data for at least 15 years, ^3^ so there is potential for misappropriation, release and misuse during this time. Genetic privacy is the protection of a person’s genetic information so they cannot be identified without knowledge and consent. ^4^ Incidental genetic findings are results unrelated to the initial reason for genetic testing. These results may have implications for the current and future health and wellbeing of participants e.g., if genetic results influence susceptibility to disease or raise questions about ancestry or parentage. In Australia, the National Health and Medical Research Council (NHMRC) has recommendations on how to address genetic topics in clinical drug trials. ^5^ For example, a separate participant information sheet and consent form (PICF) for the genetic component of studies should be used to discuss the nature of the genetic testing and its consequences for participants.

The attitudes of patients and the public to genetic testing in clinical trials have been explored in a very limited number of previous studies. These two groups generally favour the return of incidental genetic findings, ^6, 7^ but sometimes express concerns regarding genetic privacy and security, fearing disclosure of genetic information and widespread data sharing and misuse. ^8, 9^ There are no comparable data on these genetic topics for healthy volunteers. Given that healthy volunteers and patients are motivated differently for clinical trial participation, ^10, 11^ for example, healthy volunteers primarily focus on financial remuneration whilst patients do not, differences in attitudes towards genetic testing between the groups may be expected. Thus, the aim of this study was to explore the attitudes of healthy volunteers in phase 1 studies to the issues of genetic security, genetic privacy and the return of incidental genetic findings.

## Methods

### Study design

Potential participants being screened for studies at a clinical trial facility (CMAX Clinical Research Pty Ltd, Adelaide, Australia) were questioned about their attitudes to genetic testing using a paper survey (May to September 2019). No formal sample size was set, with as many potential participants approached during the study period as possible. The survey was de-identified and no personal information was collected apart from demographics. No potential biases were considered to influence participation and therefore the inclusion criteria were kept simple: participants were eligible if they were presenting for a healthy volunteer clinical drug study, were ≥18 years of age, and had sufficient English to allow informed consent and to answer the survey. There were no further inclusion or exclusion criteria. Informed consent was obtained by the lead author (S.L.) and participants completed the survey independently in the waiting room. The Human Research Ethics Committee at the University of Adelaide approved the study (H-2019-085).

### Surveys

Surveys were written by the authors to investigate attitudes towards genetic security, genetic privacy and incidental genetic findings. Questions were written in non-technical language to maximise readability and understanding. No formal validation or piloting of the survey was conducted prior to enrolment of the first participant. There were two iterations of the survey: an initial version (1 ^st^ survey) involving Q1-6 and then an updated version (2 ^nd^ survey) that included three additional questions (Q7-9) (see extended data ^12^). Several question formats were used. A 5-point Likert scale included the options “very unimportant”, “unimportant”, “neutral”, “important” or “very important”. Other questions used a rank scale method (1-5, with 1 being the least important and 5 being the most important), and in the case of ties, a mean rank was ascribed to the responses. Polar responses (yes/no) were used for statements regarding incidental genetic findings. The final question format was a visual analogue scale to investigate attitudes towards genetic privacy. The demographic data collected were age, ethnicity (2 ^nd^ version of the survey only), level of education, and the number of clinical trials undertaken previously at CMAX Clinical Research. The definitions of ethnicity were those used by the Australian Standard of Classification of Ethnic and Cultural Groups. ^13^

### Data analysis

Data from paper surveys were transcribed electronically and analysed using SPSS v25 (IBM corporation, Armonk, NY). Some responses were dichotomised as “favoured” (comprising the responses “important” and “very important”) or “not favoured” (comprising the responses “very unimportant”, “unimportant” and “neutral”). After analysis of the first 190 surveys, an amended survey was created which included three new questions. These questions created scenarios to discern the motivations for some responses. A sample size of 300 was calculated to result in a 95% confidence interval of 5-10% for a range of proportions between 30%-70% for each question. Potential statistically significant relationships between responses and demographics were examined. Kruskal-Wallis tests were performed for any relationships with age, number of previous CMAX studies, ethnicity, and education. Mann-Whitney U-tests were performed for any relationships with gender. Statistical significance was set at p < 0.05.

## Results

### Demographics

Table 1 shows the demographic results of the study. There were 275 respondents who completed both surveys (85.8% participation) – 189 for the first and 85 for the second iteration. The mean age was 27 years (range 18-73 years); 54% were male, 55% completed high school as their highest form of education, 62% had not undertaken a clinical trial at CMAX Clinical Research previously, and 53% were of North/Western European ethnicity ( Table 1). ^16, 17^

**Table 1.** Demographics.

Age		Gender		Number of CMAX studies		Education		Ethnicity	
	No.		No.		No.		No.		No.
18-22	66	Male	149	0	167	Primary School	2	North/Western Europe	45
23-27	74	Female	126	1-2	57	High School	150	Southern/Eastern Europe	8
28-32	34			3-4	21	University	92	Aboriginal & Torres Strait Islander	0
33-37	21			5+	25	Postgraduate	27	Southeast Asia	10
38-42	14							Northeast Asia	1
43-47	21							Southern/Central Asian	3
48-52	14							North African/Middle Eastern	1
53-57	14							Sub-Saharan African	2
58-62	10							Oceania	4
>62	12							Native peoples of the Americas	4
								Uncertain	6
Total	275	Total	275	Total	263	Total	271	Total	84

### Genetic security

**Figure 1.**
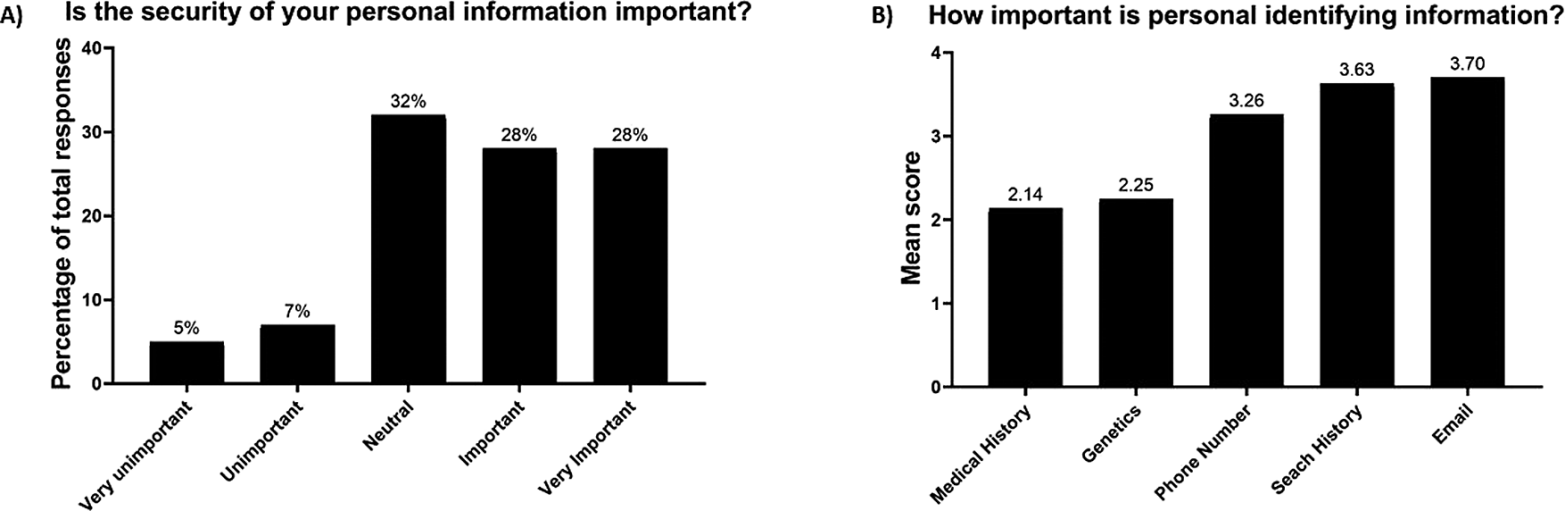
Attitudes toward the importance of genetic security (A) and the level of importance of (Scale 1-5) of personal identifying information (B).

Just over half the participants had a favourable attitude towards the importance of genetic security (56%) ( Table 2 and Figure 1A). The favourable response was significantly associated with younger age (p=0.017) and the median age of favoured responses was 26 years. When participants were asked to rank the security of their genetic information against other personal identifying information, genetics had a similar mean rank to medical history, and was ranked less important than the other personal information such as mobile phone number, internet browser search history and email address ( Figure 1B). There were no significant associations between this ranking question and demographics ( Table 2).

**Table 2.** Dichotomised results for Likert scale questions.

Question	n	Favour	Not favoured
Is genetic security important to you?	274	152(55.5%)	122(44.5%)
Is genetic privacy important to you?	274	156(56.9%)	118(43.1%)
Do you think incidental genetic findings should be mentioned in the information sheet?	275	239(86.9%)	36(13.1%)
Do you think it is important to be informed of any incidental genetic findings?	275	248(90.2%)	27(9.8%)
If your genetic information is re-analysed, would you want to be informed of these results?	190	169(88.9%)	21(11.1%)

### Genetic privacy

**Figure 2.**
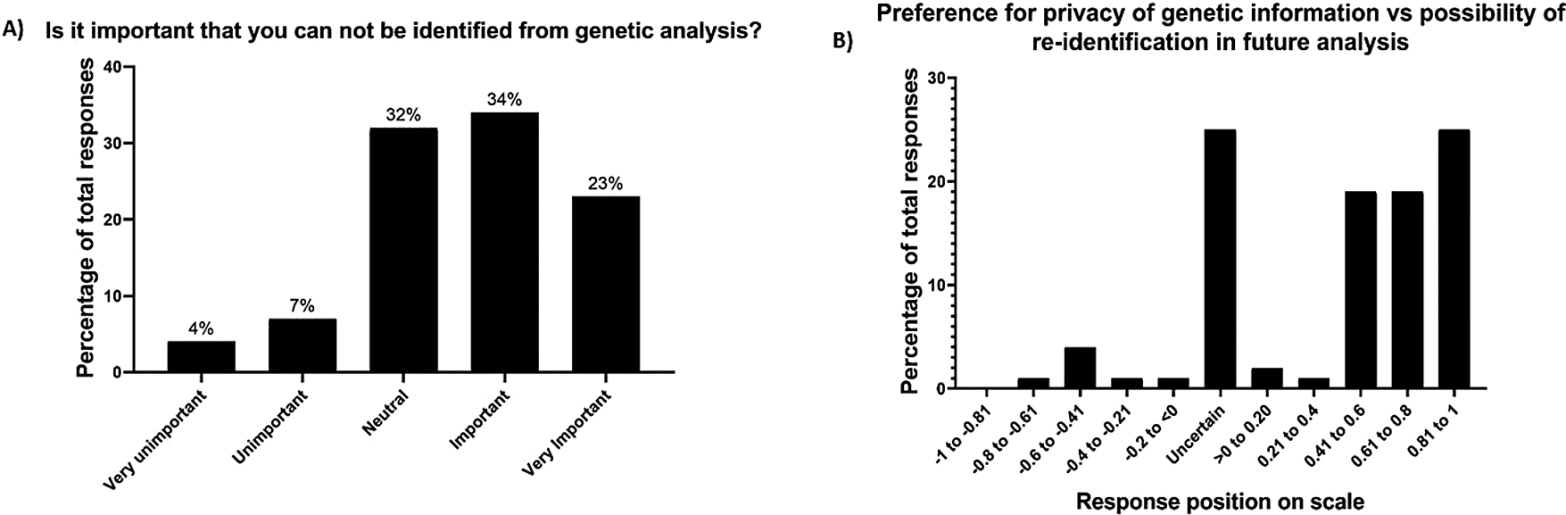
Attitudes towards the importance of genetic privacy (A) and preference for genetic privacy versus re-identifiability, where −1 is total genetic privacy and 1 is total re-identifiability (B).

More than half the participants had a favourable attitude towards the importance of genetic privacy (57%) ( Table 2 and Figure 2A). No demographic factors were associated with responses to this question. When choosing between genetic privacy and re-identifiability, most respondents (67%) chose an option in the re-identifiability portion of the visual analogue scale ( Figure 2B). Women were more likely to prefer re-identifiability than men (median on response scale of 0.70 versus 0.47, p=0.033), but no other statistical relationships to demographics were found ( Table 3).

**Table 3.** Statistical analyses.

Question		Age	Gender	Education	Number of previous studies at CMAX Clinical Research	Ethnicity
Is genetic security important to you?		p = 0.017	ns	ns	ns	ns
Is genetic privacy important to you?		ns	ns	ns	ns	ns
Do you think incidental findings should be mentioned in the information sheet?		ns	ns	ns	ns	ns
Do you think it is important to be informed of any incidental findings?		ns	ns	ns	ns	ns
If your genetic information is re-analysed, how important is it you are informed of these results?		ns	ns	ns	ns	na
What risk level would you accept for the following diseases?						
	Cancer	p = 0.035	ns	ns	p = 0.006	ns
	Heart disease	p = 0.004	ns	ns	p = 0.010	ns
	Neurodegeneration	p = 0.0001	ns	ns	p = 0.015	ns
Would you trade the privacy of your genetic information for re-identifiability in a future outcome?		ns	p = 0.033	ns	ns	ns
Rank your personal information in terms of how important the security of the information is to you		ns	ns	ns	ns	ns
If you had a 50% risk of developing a potentially fatal form of untreatable cancer would you want results returned?		ns	ns	ns	ns	ns

ns, not statistically significant; na, not applicable.

### Incidental genetic findings

**Figure 3.**
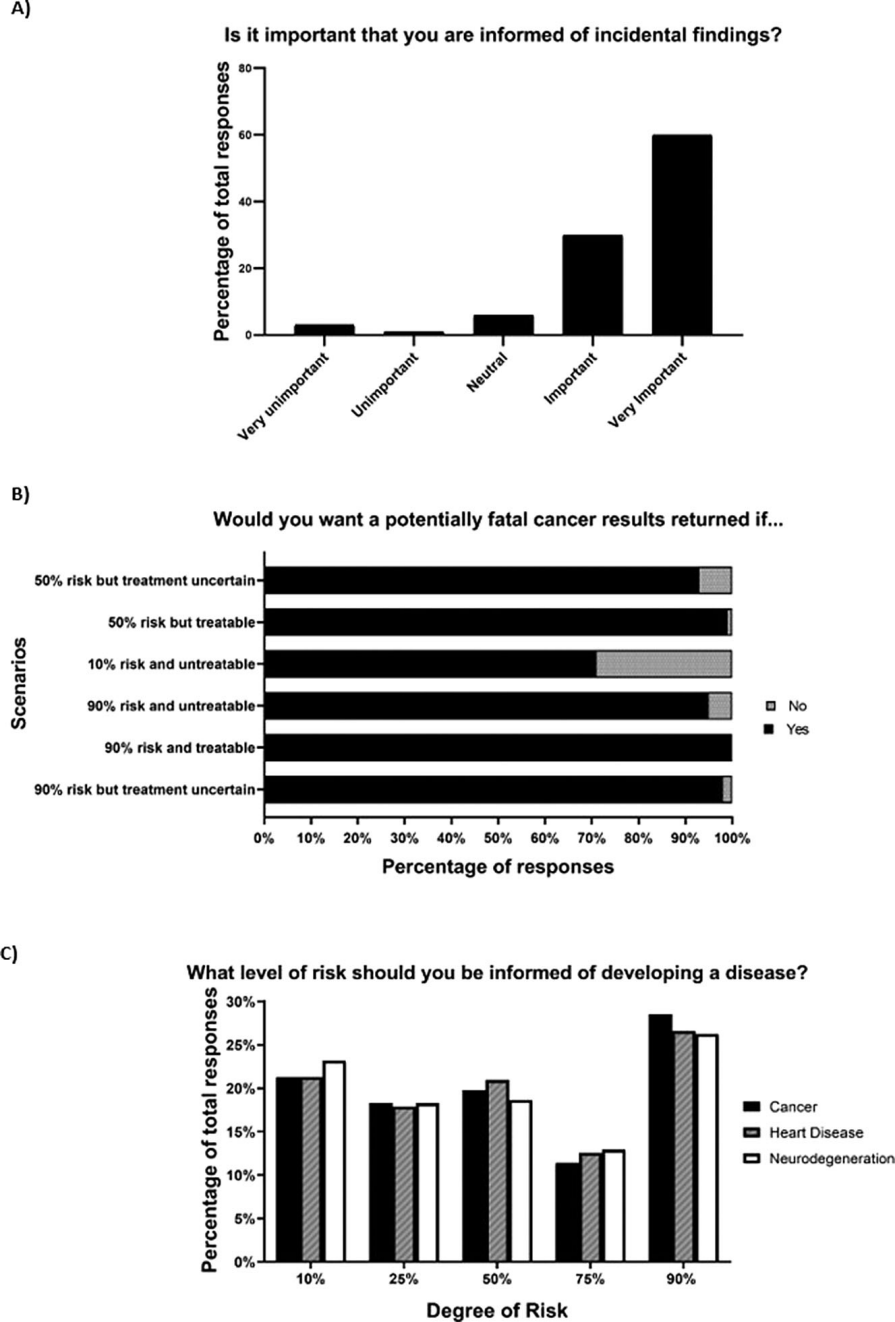
Attitudes towards the importance of returning incidental genetic findings (A), various cancer scenarios that could influence the return of incidental genetic findings (B), and the degree of risk that could influence the return of incidental genetic findings (C).

Participants strongly preferred the return of incidental genetic findings (90% favoured) and wanted this information included in PICFs (87% favoured) ( Table 2) ( Figure 3A). Both questions were not associated with any statistically significant relationships to demographics. Respondents also preferred the return of incidental genetic findings for several cancer scenarios with varying hypothetical risks and treatability, but they were slightly less interested if the risk was very low and the cancer was untreatable ( Figure 3B). When participants were asked to choose a risk for a specific disease that should trigger the return of incidental genetic findings, answers were similarly divided across all levels of risk (10-90%). The three most popular answers were for the options of 90% risk for developing a disease, with cancer > heart disease > neurodegeneration ( Figure 3C). Older participants were more likely to select higher risk of disease to trigger the return of incidental genetic findings, whereas respondents with more clinical trial experience studies wanted information provided at lower levels of risks ( Table 3). About 90% of participants wanted information provided to them in the future if sample re-analysis or new genetic discoveries could influence their health and wellbeing ( Table 2).

## Discussion

This is the first study to report the attitudes of healthy volunteers to genetic testing in phase 1 clinical trials. These attitudes are important to understand given the rise of genetic testing in early drug development and the unique ethical and practical aspects of testing healthy volunteers who typically have few interactions with healthcare providers. Here, the focus was the genetic testing issues previously explored in limited studies with patients and the public – genetic security, genetic privacy, and the return of incidental genetic findings. ^6– 11^

There was relative indifference towards genetic security, with healthy volunteers ranking the importance of this topic lower than for other personal information. Indeed, mobile phone number and email address are provided to the clinical trial site during screening, so for genetic security to rank lower than these two pieces of information emphasises the relative indifference to the topic. This is similar to limited data in patients, with only 35% of potential biobank participants and just one patient in a focus group of 15 with epilepsy expressing concerns about genetic security. ^8, 14^

Regarding genetic privacy, healthy volunteers also held mixed opinions, with just over half considering it “important” or “very important”. This is comparable to a survey of 4659 US adults from the public, in which 44% would protect genetic test results, whereas the others were happy to share their genetic data with researchers. ^15^ Additionally, a survey assessing the views of 57 patients to pharmacogenomic testing showed that approximately 40% expressed concerns about privacy. ^9^ When asked whether they would trade privacy for re-identifiability in the future, respondents to our survey overwhelmingly favoured re-identifiability of their genetic information. Taken together, these findings support clinical research governance that de-identifies genetic information to maintain privacy, but with mechanisms in place to re-identify that information if asked by clinical trial participants.

The healthy volunteers wanted incidental genetic findings returned to them, and this applied to a range of hypothetical clinical scenarios with varying degrees of disease risk. Older participants and those with more clinical trial experience accepted higher disease risk before triggering the return of such information. Exactly why these two groups indicated higher disease risk is unclear, but it may be simply related to life experience and greater acceptance of the unknown. The majority of oncology patients in a previous study also favoured the return of incidental findings, even if they constituted “bad news”, such as high risk of developing an untreatable cancer. ^6^ Studies of the public indicate the same (4961 participants), with “treatability” being an important consideration and with 98% in favour of findings being returned for “a serious disease that is life threatening but could be prevented”. ^7^ Together with the results in healthy volunteers, these data suggest that everyone should be asked about their wishes for the return of incidental genetic findings when being screened for clinical trials. ^5^

There are several limitations of the study. First, it was conducted at one site, so care is required in extrapolating the results to other phase I clinical units with significantly different volunteer demographics. Second, the survey was offered opportunistically to all people in the waiting room, independent of the type of clinical trial screening. It is possible that attitudes to genetic testing are different between healthy volunteers being screened for clinical trials with genetic testing and those being screened for clinical trials without genetic testing. Third, the study did not assess baseline “genetic literacy”, so misunderstanding of questions is possible, particularly the conceptually more difficult questions related to disease risks. Lastly, the study was not designed to capture the nuances of genetic testing because the topic is too broad and complex to be condensed into a short survey e.g., the specifics of testing.

In conclusion, healthy volunteers who are screening for phase 1 clinical trials have mixed views about the importance of genetic security and genetic privacy, but they strongly favour the return of incidental genetic findings that could affect their health. These issues should be discussed with potential participants during informed consent for phase 1 clinical trials with genetic testing.

## Data availability

### Underlying data

Figshare: Attitudes to Genetic Testing in Phase 1_Survey Data. https://doi.org/10.6084/m9.figshare.14204507.v1. ^16^

Figshare: Untitled Attitudes to Genetic Testing in Phase 1_Amalgamated Survey Data. https://doi.org/10.6084/m9.figshare.14204516.v1. ^17^

### Extended data

Figshare: Attitudes to genetic testing in phase 1 survey questions. https://doi.org/10.6084/m9.figshare.14182898.v1. ^12^

This project contains the following extended data:

- A supplementary figure showing all the survey questions used in the study.

Data are available under the terms of the Creative Commons Attribution 4.0 International license (CC-BY 4.0).
