# Supplementary material for: Attitudes of healthy volunteers to genetic testing in phase 1 clinical trials
Source: F1000Res. 2021 Mar 30;10:259. [Version 1] doi: 10.12688/f1000research.26828.1 (PMC8787555; doi:10.12688/f1000research.26828.1)
Supplement: Peer review history

# Peer review: Reviewer response for version 1

**DOI:** 10.5256/f1000research.29624.r119428

**Published:** 2022-01-24

**Authors:** Colleen Aldous

**Affiliations:** 1 College of Health Sciences, Nelson R. Mandela School of Clinical Medicine, University of KwaZulu-Natal, Durban, South Africa

Healthy participants were selected to complete an unpiloted and non-validated questionnaire which investigated attitudes to genetic information security, privacy and the return of incidental genetic findings. Ethics permission was provided and informed consent was noted. This is a descriptive study and the data is presented appropriately. The Lickert scale data is converted into a form which can be statistically compared. The findings are clearly summarised. There is a conclusion based on the data that the return of incidental genetic findings should be addressed. The limitations of the study are stated.

 Queries:

- A sample size of 300 participants had been calculated, however the final number reported on was 275. Could the authors please clarify this point?
- Ethnicity is addressed but seems not to have much of a role in the answering of the research questions. In the abstract the reader is left questioning the other ethnicities after only North/Western European ethnicity is mentioned. In Table 1 the ethnicity of only 84 participants appears to have been reported. I would suggest that the authors state that ethnicity, for this study, was not addressed. 

This study raises further questions for future studies.

Is the work clearly and accurately presented and does it cite the current literature?

Partly

If applicable, is the statistical analysis and its interpretation appropriate?

Yes

Are all the source data underlying the results available to ensure full reproducibility?

Yes

Is the study design appropriate and is the work technically sound?

Partly

Are the conclusions drawn adequately supported by the results?

Yes

Are sufficient details of methods and analysis provided to allow replication by others?

Yes

Reviewer Expertise:

Healthcare research with a special interest in rare and congenital disorders.

I confirm that I have read this submission and believe that I have an appropriate level of expertise to confirm that it is of an acceptable scientific standard.

# Peer review: Reviewer response for version 1

**DOI:** 10.5256/f1000research.29624.r82525

**Published:** 2021-04-06

**Authors:** Martin D. Lewis

**Affiliations:** 1 South Australian Health and Medical Research Institute, Adelaide, SA, Australia; 2 College of Medicine and Public Health, Flinders University, Adelaide, SA, Australia

The authors address important aspects of genetic testing, namely participant concerns regarding data security, privacy and incidental findings. As most participants were found to favour being informed of incidental findings, this should influence future consent options for study participants. The number of studies involving genetic testing is increasing, with this fundamental component of the ethics process critically important.

 The article is well written, with the study, survey design, and analysis very clearly and appropriately described. The literature cites the current national guidelines and standards, in addition to relevant publications in the manuscript focusing on questions addressed in this field. All data was made available ensuring reproducibility. The conclusions draw from the data are adequately supported.

**Minor changes:**

 Page 4 - The sentence under Demographic stating, “There were 275 respondents that completed both surveys…” could be misunderstood as some respondents completed two surveys. This could be altered to “There was a total of 275 respondents that completed the first (189) or second (85) iteration of the survey”. 

 There appears to be an inconsistency with the previous paragraph under ‘Data Analysis’ that says after analysis of the first 190 surveys, a second iteration was created. Were 190 survey of the first iteration started and only 189 completed?

Is the work clearly and accurately presented and does it cite the current literature?

Yes

If applicable, is the statistical analysis and its interpretation appropriate?

Yes

Are all the source data underlying the results available to ensure full reproducibility?

Yes

Is the study design appropriate and is the work technically sound?

Yes

Are the conclusions drawn adequately supported by the results?

Yes

Are sufficient details of methods and analysis provided to allow replication by others?

Yes

Reviewer Expertise:

Genetics, neuroscience, cell biology, major depressive disorder and pharmacogenomics.

I confirm that I have read this submission and believe that I have an appropriate level of expertise to confirm that it is of an acceptable scientific standard.
